# Acute Corneal Hydrops Mimicking Infectious Keratitis as Initial Presentation of Keratoconus in a 10-Year-Old Child

**DOI:** 10.1155/2015/308348

**Published:** 2015-03-31

**Authors:** Elise A. Slim, Elias F. Jarade, Bilal M. Charanek, Joelle S. Antoun, Adib I. Hemade, Sahar H. Awada, Henry W. Fakhoury, Carole G. Cherfan

**Affiliations:** ^1^Beirut Eye Specialist Hospital, Al-Mathaf Square, P.O. Box 116-5311, Beirut, Lebanon; ^2^Saint Joseph University Hospital, Faculty of Medicine, P.O. Box 166830, Beirut, Lebanon; ^3^Mediclinic, Dubai Mall, Dubai, UAE; ^4^Lebanese University, Rafic Hariri University Campus, P.O. Box 6573/14, Hadath, Beirut, Lebanon

## Abstract

*Purpose*. To report a case of acute hydrops in a 10-year-old child with advanced keratoconus. *Case Presentation*. A ten-year-old boy diagnosed as having right eye (RE) infectious keratitis, not responding to antimicrobial therapy, was referred to our hospital. The diagnosis of infectious keratitis was established one month prior to his presentation following an episode of acute corneal whitening, pain, and drop in visual acuity. Topical fortified antibiotics followed by topical antiviral therapy were used with no improvement. Slit lamp examination showed significant corneal protrusion with edema surrounding a rupture in Descemet's membrane in the RE. The diagnosis of acute corneal hydrops from advanced keratoconus was highly suspected and confirmed with corneal topography. *Conclusion*. Although a relatively rare disease at the age of 10 years, keratoconus can be rapidly progressive in the pediatric group. Keratoconus should always be considered in the differential diagnosis of progressive vision loss in this age group.

## 1. Introduction

Keratoconus (KC) is a noninflammatory ectasia of the cornea. Classically, the onset of KC is during puberty and the condition is progressive until the third or fourth decade of life [1]. In fact, KC demonstrates an increased incidence and faster progression at both puberty and pregnancy due to hormonal influences [2, 3]. A cross-sectional study of 482 eyes found that keratoconic eyes in patients younger than 40 years of age had a 10 times higher rate of severe disease as compared to older age groups [4]. Another case control study found that patients aged 30 or younger conferred a sevenfold increased risk of corneal transplantation in KC patients as compared with patients older than 30 years of age. Experimental studies have shown an age-related change in corneal collagen fibril properties that might contribute to an increase in stiffness of the cornea with age [5]. However, most of the studies report on KC starting from adolescence, with very few reports in the prepubertal age group. Pediatric KC is a rare entity that is often overlooked or misdiagnosed. Moreover, in otherwise healthy children, eye rubbing seems to be the only factor associated with increased incidence and progression of KC [6–11]. Peculiarly, KC in this age group can be rapidly progressive which may lead to permanent visual disability in case of delayed treatment. Herein, we present the case of acute corneal hydrops as the initial presentation of KC in a 10-year-old child that was misdiagnosed as infectious keratitis.

## 2. Case Report

**Figure 1:**
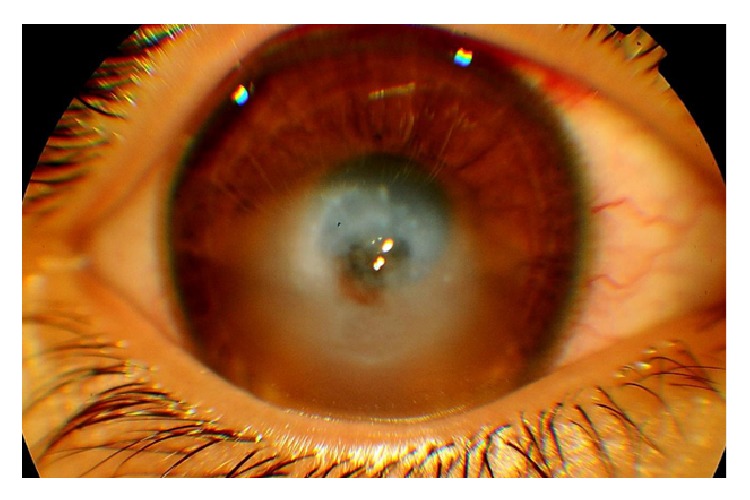
Slit lamp exam of the right eye showing a central Descemet's membrane rupture with inferocentral corneal edema (hydrops).

A ten-year-old boy diagnosed as having right eye (RE) infectious keratitis, not responding to antimicrobial therapy, was referred to our eye hospital for further management. The diagnosis of infectious keratitis was established one month prior to his presentation in an outside institution following an episode of acute corneal whitening, pain, and drop in visual acuity in his RE. A regimen of topical fortified antibiotics was implemented followed by a regimen of topical antiviral therapy with no improvement. The patient had a history of eye rubbing and progressive visual loss over the past year. However, no previous ocular examination was performed. At the time of presentation, the visual acuity in the RE was counting fingers near face (not improving with refraction) and the best-corrected vision in the left eye (LE) was 20/25 with a refraction of −6.75 + 4.75 × 55. The retinoscopy exam showed scissoring in the LE. However, retinoscopy could not be performed in the RE due to a poor red reflex. The slit lamp examination showed significant corneal protrusion with edema surrounding a rupture in Descemet's membrane in the inferior midportion of the cornea in the RE (Figure 1). The cornea in the LE was clear. Anterior chamber was calm with no signs of infection. In both eyes, examination of the superior palpebral conjunctivae demonstrated mild generalized hyperaemia and a moderate papillary response, which were indicative of allergic conjunctivitis. Dilated ocular fundus examination confirmed normal posterior segment in the LE and was not visible in the RE.

**Figure 2:**
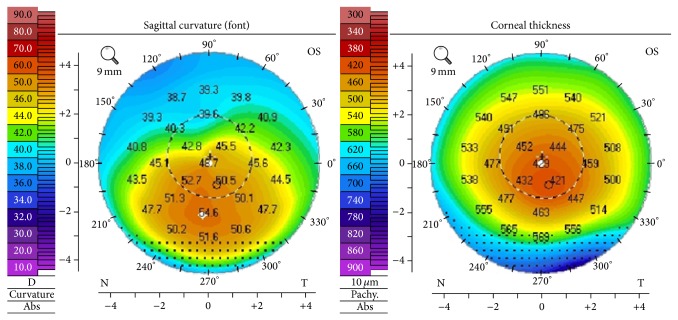
Corneal topography of the left eye showing stage II keratoconus.

Based on clinical findings, acute corneal hydrops from advanced KC in the RE was highly suspected. Asymmetrical KC was confirmed with corneal topography (Pentacam 70700, Oculus, Germany) which revealed the presence of advanced KC in the RE and stage II KC (Amsler-Krumeich classification) in the LE (Figure 2). To be noted, central corneal thickness was significantly reduced in the LE to 418 um with an inferiorly positioned corneal apex, consistent with KC.

## 3. Discussion

In this case, acute corneal hydrops was the initial presentation of KC in a pediatric patient with a suggestive history of allergic conjunctivitis, eye rubbing, and progressive loss of vision. Corneal hydrops was misdiagnosed as infectious keratitis, and KC was overlooked. This may have been due to the infrequency of KC during childhood but was most likely due to the rarity of occurrence of hydrops in this age group. Acute corneal hydrops is the development of a break in Descemet's membrane with subsequent marked edema of the corneal stroma and epithelium [6]. It is well known to occur in corneal ectasia. However, it is rather infrequent in KC whereby it occurs in only 3% of patients [12, 13]. Although usually self-limiting, it often leaves a vision-impairing scar and leads to serious ocular complications. The mean age at the onset of corneal hydrops was 39.3 years in one study [12] and 24 years in another [14]. Many risk factors for the development of corneal hydrops in KC were reported and they include childhood diagnosis of KC, male sex, poor corrected visual acuity at the diagnosis of KC, and severe allergic eye disease [12, 15]. In our reported case, besides the early onset of KC and the male sex, allergic eye disease with eye rubbing may have played a major role in the development of corneal hydrops in KC in this young age group. Hence, in our case, allergic eye disease and eye rubbing may have contributed to the development of KC at this early age and eventually lead to the development of acute hydrops. The hypothesis that eye rubbing is the most significant cause of KC is supported by many reports and dates back to 1956 [16–19]. Moreover, several case reports link eye rubbing to the development of acute hydrops in KC [14, 19, 20]. The infrequency of KC during childhood and the rarity of occurrence of acute hydrops in this age group are supported by the paucity of reports on pediatric KC in the literature with only two cases of acute corneal hydrops in children previously reported [8, 9]. To the best of our knowledge, this is the third reported case of acute corneal hydrops in the pediatric population. Downie reported a case of bilateral corneal hydrops in an 8-year-old boy with atopic disease [9], and Panahi-Bazaz et al. reported another case of acute bilateral hydrops in a 7-year-old girl with vernal keratoconjunctivitis [8]. Similar to our case, acute corneal hydrops was the first presentation of KC in the 2 reported cases with both patients diagnosed with allergic conjunctivitis and eye rubbing, further emphasizing the fact that allergic keratoconjunctivitis with eye rubbing may increase the incidence of corneal hydrops in children with KC [13, 21, 22]. Unlike the previously reported cases, the hydrops in our case was initially misdiagnosed despite its reoccurrence and resistance to treatment for infectious keratitis. The diagnosis was not made until one month after acute corneal hydrops had occurred. This late diagnosis predisposes children to serious complications of corneal hydrops including corneal perforation, microbial keratitis, glaucoma, and amblyopia. Hence, corneal leukoma should arouse the suspicion of acute hydrops even in children. It is of note that other environmental or genetic factors may have played a role in the development and progression of KC in otherwise healthy children. Many studies have shown that in the Middle East patients present with severe KC at a much younger age than in western populations and have a higher incidence of associated atopic eye disease [21, 22]. Thus, the presence of allergic eye disease in children in our region should raise the index of suspicion of associated KC.

In conclusion, in this case, acute hydrops was the initial clinical presentation of advanced KC in a 10-year-old pediatric patient previously misdiagnosed as infectious keratitis. Although it is a relatively rare disease at the age of 10 years, pediatric KC can be rapidly progressive especially in the presence of allergic conjunctivitis and eye rubbing. This entity should always be considered in the differential diagnosis of progressive vision loss and of corneal leukoma in this young age group. Moreover, this case signals the fact that children with atopia should always be referred to the ophthalmologists for regular eye exams. In addition, a high index of suspicion for progressive KC should always be apprehended especially when it is associated with progressive vision loss.
